# Appetite-regulating hormone trajectories and relationships with fat mass development in term-born infants during the first 6 months of life

**DOI:** 10.1007/s00394-021-02533-z

**Published:** 2021-03-25

**Authors:** Kirsten S. de Fluiter, Gerthe F. Kerkhof, Inge A. L. P. van Beijsterveldt, Laura M. Breij, Leonie C. van Vark-van der Zee, Monique T. Mulder, Marieke Abrahamse-Berkeveld, Anita C. S. Hokken-Koelega

**Affiliations:** 1 Department of Pediatrics, Subdivision of Endocrinology, Erasmus University Medical Center-Sophia Children’s Hospital, Room SK-0150, Wytemaweg 80, 3015 CN Rotterdam, The Netherlands; 2 Dutch Growth Research Foundation, Rotterdam, The Netherlands; 3 Section of Pharmacology Vascular and Metabolic Diseases, Department of Internal Medicine, Cardiovascular Research School COEUR, Erasmus University Medical Center, Rotterdam, The Netherlands; 4 Danone Nutricia Research, Utrecht, The Netherlands

**Keywords:** Infants, Appetite-regulating hormones, Body composition, Adiposity, Macronutrients

## Abstract

**Background:**

The first 6 months of life are a critical window for adiposity programming. Appetite-regulating hormones (ARH) are involved in food intake regulation and might, therefore, play a role in adiposity programming. Studies examining ARH in early life are limited.

**Purpose:**

To investigate ghrelin, peptide YY (PYY) and leptin until 6 months and associations with fat mass percentage (FM%), infant feeding and human milk macronutrients.

**Procedures:**

In 297 term-born infants (Sophia Pluto Cohort), ghrelin (acylated), PYY and leptin were determined at 3 and 6 months, with FM% measurement by PEAPOD. Exclusive breastfeeding (BF) was classified as BF ≥ 3 months. Human milk macronutrients were analyzed (MIRIS Human Milk Analyzer).

**Main findings:**

Ghrelin increased from 3 to 6 months (*p* < 0.001), while PYY decreased (*p* < 0.001), resulting in increasing ghrelin/PYY ratio. Leptin decreased. Leptin at 3 months was higher in girls, other ARH were similar between sexes. Leptin at 3 and 6 months correlated with FM% at both ages(*R* ≥ 0.321, *p* ≤ 0.001) and gain in FM% from 1 to 6 months(*R* ≥ 0.204, *p* = 0.001). In BF infants, also ghrelin and ghrelin/PYY ratio correlated with this gain in FM%. Exclusively BF infants had lower ghrelin and higher PYY compared to formula fed infants at 3 months (*p* ≤ 0.039). ARH did not correlate with macronutrients.

**Conclusions:**

Increasing ghrelin and decreasing PYY, thus increasing ghrelin/PYY ratio, suggests an increasing orexigenic drive until 6 months. ARH were different between BF and FF infants at 3 months, but did not correlate with human milk macronutrients. Ghrelin and leptin, but not PYY, correlated with more FM development during the first 6 months, suggesting that they might be involved in adiposity programming.

**Supplementary Information:**

The online version contains supplementary material available at 10.1007/s00394-021-02533-z.

## Introduction

Appetite-regulating hormones (ARH) are involved in the regulation of food intake through specific brain centers, such as the hypothalamus that plays a key role in controlling glucose, energy homeostasis and food intake [1, 2]. Ghrelin and peptide YY (PYY) are secreted from the gastrointestinal tract [2]. Leptin is secreted mainly from adipose tissue [1, 3] and also from the stomach, but systemic effects of gastric leptin are negligible [4]. Ghrelin stimulates food intake; whereas, PYY and leptin decrease appetite and increase metabolic rate [2, 3]. In addition, the ghrelin/PYY ratio is of interest as a marker of orexigenic drive, rather than ghrelin and PYY levels separately [5, 6].

Early life rapid weight gain, and specifically during the first months of life, has been associated with an increased adiposity and cardiovascular disease risk in adulthood [7–11]. In addition, we have shown that particularly the change in fat mass percentage (FM%) during the first 6 months, in contrast to the 6- to 12-month period, is associated with higher FM% and abdominal subcutaneous FM at the age of 2 years [12]. These first 6 months after birth are considered a critical window for adiposity programming [9, 10], ARH trajectories might be of importance in unraveling this early adiposity programming. ARH have been associated with later growth and adiposity, but most studies used cord blood [13–18] or newborn blood spots [19, 20] to investigate ARH at birth or in specific groups (such as infants born premature or small-for-gestational age) [21, 22]. However, data on ghrelin, PYY and leptin trajectories during early life in healthy term-born infants are very limited.

Few studies have compared ARH levels between breast-fed (BF) and formula-fed (FF) infants in early life. Two studies investigated ghrelin and leptin levels during the first four months [23, 24]. Our group reported differences in ghrelin, PYY and leptin levels between BF and FF infants at age 3 months [25]. Human milk macronutrient composition could potentially influence appetite-regulating hormone levels in BF infants as we previously found that exclusively BF infants-receiving human milk with higher fat and energy were satiated earlier. This could be a self-regulatory mechanism to prevent intake of excessive macronutrients [26]. Associations between ARH and human milk macronutrients and infant appetite until age 6 months, a critical window for adiposity programming as mentioned above, are lacking.

The primary objective of this study was to investigate ghrelin, PYY and leptin levels during the first 6 months of life and their associations with body fat mass development. The other objectives were to investigate ARH in association with infant feeding, human milk macronutrients and appetite. We hypothesized that higher ghrelin and leptin levels would associate with a higher gain in FM% during the first 6 months. Furthermore, we hypothesized ARH levels would be different between BF and FF infants and that ghrelin would be lower and PYY and leptin levels higher in infants-receiving human milk with a higher fat and energy content.

## Materials and methods

### Study setting and subjects

The study population consisted of healthy, term-born infants, participating in the Sophia Pluto Study, a birth cohort study in Rotterdam area (The Netherlands). All infants fulfilled the following inclusion criteria: term-born (≥ 37 weeks of gestation), age < 28 days, uncomplicated neonatal period without signs of severe asphyxia (defined as an Apgar score < 3 after 5 min) and no sepsis or long-term complication of respiratory ventilation. Infants were excluded if they had known congenital or postnatal diseases, confirmed intrauterine infection, maternal use of corticosteroids during pregnancy, or a significant maternal medical condition, such as (gestational) diabetes, that could interfere with the study results. The Sophia Pluto Study obtained approval by the Medical Ethics Committee of Erasmus Medical Center and parental written informed consent for every participant. For present study, we included 297 singleton-born infants from whom blood samples were obtained at age 3 months regardless of infant feeding type.

### Data collection and measurements

Outpatient clinic visits were scheduled at the age of 1, 3 and 6 months (Table 1). Pregnancy and birth data were obtained from midwife and hospital records. Measurements and blood collection were performed by trained staff.

**Table 1:** Clinical characteristics of the study population (*n* = 297 infants)

	Boys	Girls	*p* value
Birth			
Weight (kg)	3.38 [3.12–3.77]	3.27 [2.94–3.71]	**0.022**
Length (cm)	51.0 [49.0–52.0]*	50.0 [48.0–51.0]*	**0.013**
FM (%)	NA	NA	
1 month			
Weight (kg)	4.37 [4.02–4.73]	4.08 [3.64–4.40]	** < 0.001**
Length (cm)	55.0 [53.5–56.5]	54.0 [52.1–55.4]	**0.003**
FM (%)	16.1 [13.5–18.9]	16.0 [13.6–19.4]	0.32
3 months			
Weight (kg)	6.18 [5.73–6.64]	5.73 [5.15–6.18]	** < 0.001**
Length (cm)	62.0 [60.5–63.2]	60.5 [58.9–61.5]	0.26
FM (%)	22.1 [19.0–24.6]	23.3 [19.6–26.5]	0.099
6 months			
Weight (kg)	7.87 [7.34–8.45]	7.33 [6.82–7.76]	** < 0.001**
Length (cm)	68.9 [67.0–70.2]	67.0 [65.1–68.3]	** < 0.001**
FM (%)	23.5 [20.0–26.7]	25.1 [21.6–28.9]	**0.036**

Bold significance represent by *p* < 0.05

Data expressed as median [IQR] for boys and girls

*FM* fat mass, *NA* not applicable

*Birth length available for 94 boys and 80 girls

### Anthropometrics

Weight was measured to the nearest 5 g by an electronic infant scale (SECA 717, Hamburg, Germany). Length was measured twice by two-person technique to the nearest 0.1 cm with an infantometer (SECA 416). Birthweight standard deviation scores (SDS) were calculated [27] using Growth Analyser (https://growthanalyser.org/).

### Blood samples

At age 3 months, 297 blood samples were collected by toe prick after the infants had fasted for a minimum of 2 h. For 184 of these infants, we also collected a blood sample at age 6 months. This number was less than at 3 months because infants were either too distressed to allow a blood collection or had not yet reached age 6 months. Blood samples were collected in EDTA tubes and DPP4 (dipeptidyl peptidase-4) inhibitor, Serine Protease inhibitor and Protease inhibitor (all Merck Chemicals Netherlands—Merck KGaA) were added for stabilizing the appetite-regulating hormones. Blood was centrifuged at 4 °C to prepare plasma, which was quickly frozen and stored at − 80 °C until analyses. Ghrelin (acylated), PYY and leptin levels in plasma were determined by the MILLIPLEX MAP Human Metabolic Hormone Magnetic Bead Panel, catalog number HMHEMAG-34 K (Millipore Corporation, Billerica, MA) using the commercial protocol provided by the supplier. The intra-assay CV was 10%, and the inter-assay CV was 15%. Fasting time was calculated as time of blood collection minus time of last feeding.

### Body composition measurements

Body composition was assessed by air-displacement plethysmography (ADP by PEA POD, COSMED, Italy) as described in detail elsewhere [28]. The PEA POD was calibrated daily according to standard protocol [29].

### Infant feeding

To investigate ARH levels at age 3 months based on exclusive feeding type, infant feeding was classified as exclusive breastfeeding (BF, *n* = 158) or exclusive formula feeding (FF, n = 89) if infants received either BF or FF, respectively, and no mixed feeding for 3 months after birth.

### Breastmilk samples

Breastfeeding mothers were instructed to collect hind milk samples, thus after their infants were breastfed, at infant’s age of 3 months as described before [26]. For 61 exclusively breastfed infants, human milk samples were analyzed for macronutrient composition (fat, energy, carbohydrate and protein).

### Baby Eating Behavior Questionnaires (BEBQ)

At age 3 months, mothers were asked to fill out the Baby Eating Behavior Questionnaire (BEBQ) to assess infant appetite [30]. Each item was answered using a five-point Likert frequency scale (1 = never, 2 = rarely, 3 = sometimes, 4 = often and 5 = always).

### Statistical analysis

Clinical characteristics are expressed as median and interquartile range [IQR]. Differences in clinical characteristics were determined by independent sample Student’s *t* test or by Mann–Whitney *U* test for non-parametric parameters. ARH levels at age 3 and 6 months were analyzed using mixed model analysis. To investigate differences in ARH between boys and girls, we used sex as a covariate in the mixed models. Time was modeled by entering hospital visits at ages 3 and 6 months into the linear mixed models. Linear correlations were determined by Spearman for non-parametric parameters.

Correlations between ARH levels and human milk macronutrients were performed in infants with exclusive breastfeeding. SPSS statistical package version 25 (SPSS Inc. Chicago, Illinois) was used and *p* values < 0.05 were considered statistically significant.

## Results

Clinical characteristics of the subjects are presented in Table 1. The total group consisted of 53.5% boys. Median gestational age was 39.9 [38.9–40.7] weeks. Median infant birthweight SDS was − 0.15 [− 0.89 to 0.48], maternal pre-pregnancy BMI 23.5 [21.4–26.2] kg/m^2^ and maternal weight gain during pregnancy 14.0 [10.0–18.0] kg.

### Ghrelin, PYY and leptin levels during the first 6 months of life

Ghrelin (acylated) levels increased from age 3–6 months (*p* < 0.001), while PYY levels decreased (*p* < 0.001), resulting in an increase in ghrelin/PYY ratio over time (*p* < 0.001). Leptin levels decreased from age 3–6 months (*p* < 0.001) (Table 2).

**Table 2:** Ghrelin, PYY, Ghrelin/PYY ratio and leptin levels at age 3 and 6 months in the total group, boys and girls

	3 months				6 months			
	Total group, *n* = 297	Boys, *n* = 159	Girls, *n* = 138	*p* value	Total group, *n* = 297	Boys, *n* = 159	Girls, *n* = 138	*p* value
Ghrelin (pg/ml)	67.2 [60.9–73.5]	63.4 [54.8–71.9]	71.7 [62.4–80.9]	0.20	121.9 [107.0–136.7]	127.7 [109.3–146.2]	108.8 [83.9–133.6]	0.23
PYY (pg/ml)	230.8 [219.5–242.0]	228.4 [213.0–243.9]	233.5 [216.9–250.0]	0.66	172.3 [165.0–179.6]	173.8 [164.6–182.9]	169.4 [157.0–181.8]	0.58
Ghrelin/PYY ratio	0.35 [0.31–0.39]	0.33 [0.28–0.38]	0.37 [0.31–0.42]	0.36	0.80 [0.69–0.91]	0.83 [0.70–0.97]	0.73 [0.55–0.92]	0.39
Leptin (pg/ml)	1492.9 [1356.5–1629.2]	1306.9 [1123.0–1490.9]	1707.1 [1509.6–1904.6]	**0.004**	881.1 [742.3–1020.0]	848.1 [673.5–1022.7]	892.0 [659.3–1124.7]	0.77

Bold significance represent by *p* < 0.05

Data expressed as estimated marginal means [95% confidence interval] for the total group, boys and girls. *p* values are presented for the difference between boys and girls

*N* number, *PYY* peptide YY

Ghrelin and PYY levels and ghrelin/PYY ratio at age 3 and 6 months were not different between boys and girls (Table 2). Leptin levels at age 3 months were higher in girls compared to boys (1707.1 vs 1306.9 pg/ml, *p* = 0.004), but similar at age 6 months.

Median fasting time was 3:00 [2:25–3:35] hours at age 3 months and 2:45 [2:00–3:40] hours at age 6 months. Ghrelin levels at age 6 months correlated with median fasting time (*R* = 0.205, *p* = 0.005), while PYY levels at age 6 months correlated inversely (*R* = − 0.211, *p* = 0.004). Ghrelin/PYY ratio at age 6 months correlated with fasting time (*R* = 0.236, *p* = 0.001), but leptin levels did not correlate. Median fasting time was not different between boys and girls at age 3 and 6 months (*p* = 0.13 and 0.14, respectively).

Neither ghrelin, PYY and ghrelin/PYY ratio, nor leptin levels at age 3 and 6 months correlated with infant birthweight SDS, maternal pre-pregnancy BMI and maternal weight gain during pregnancy.

### Correlations between ARH and body fat mass during the first 6 months of life

In the total group, regardless of infant feeding type, leptin at age 3 months correlated with FM% at age 3 and 6 months (*R* = 0.395, *p* < 0.001 and *R* = 0.321, *p* < 0.001, respectively) and with the gain in FM% from 1 to 6 months (*R* = 0.204, *p* = 0.001) (Table 3). Leptin at age 6 months also correlated with FM% at age 6 months (*R* = 0.337, *p* < 0.001) and the gain in FM% from 1 to 6 months (*R* = 0.207, *p* = 0.006). Ghrelin at age 3 months correlated only with FM% at 6 months, while PYY and ghrelin/PYY ratio did not correlate.

**Table 3:** Correlations between appetite-regulating hormones and FM% at age 3 and 6 months and the gain in FM% from 1 to 6 months in the total group, breastfed infants and formula-fed infants

	Total group *n* = 297			Breastfeeding *n* = 158			Formula feeding *n* = 89		
	FM% 3 months	FM% 6 months	Δ FM% 1–6 months	FM% 3 months	FM% 6 months	Δ FM% 1–6 months	FM% 3 months	FM% 6 months	Δ FM% 1–6 months
3 months									
Ghrelin	0.003, *p* = 0.97	0.122, *p* = 0.043	0.103, *p* = 0.091	− 0.072, *p* = 0.37	0.128, *p* = 0.12	0.172, *p* = 0.037	− 0.011, *p* = 0.92	0.034, *p* = 0.77	− 0.033, *p* = 0.78
PYY	− 0.019, *p* = 0.75	0.064, *p* = 0.29	0.069, *p* = 0.26	− 0.083, *p* = 0.30	− 0.103, *p* = 0.21	− 0.104, *p* = 0.21	− 0.010, *p* = 0.93	0.214, *p* = 0.057	0.324, *p* = 0.004
Ghrelin/PYY ratio	− 0.007, *p* = 0.91	0.076, *p* = 0.21	0.059, *p* = 0.34	− 0.039, *p* = 0.63	0.154, *p* = 0.059	0.203, *p* = 0.014	− 0.039, *p* = 0.72	− 0.060, *p* = 0.60	− 0.181, *p* = 0.12
Leptin	0.395, *p* < 0.001	0.321, *p* < 0.001	0.204, *p* = 0.001	0.354, *p* < 0.001	0.320, *p* < 0.001	0.224, *p* = 0.007	0.404, *p* < 0.001	0.284, *p* = 0.011	0.187, *p* = 0.10
6 months									
Ghrelin		− 0.031, *p* = 0.68	− 0.017, *p* = 0.82		− 0.098, *p* = 0.35	− 0.078, *p* = 0.47		− 0.052, *p* = 0.69	0.111, *p* = 0.41
PYY		− 0.032, *p* = 0.66	0.100, *p* = 0.19		− 0.130, *p* = 0.22	-0.015, *p* = 0.89		0.125, *p* = 0.34	0.172, *p* = 0.20
Ghrelin/PYY ratio		− 0.019, *p* = 0.80	− 0.044, *p* = 0.56		− 0.041, *p* = 0.70	− 0.073, *p* = 0.50		− 0.066, *p* = 0.62	0.065, *p* = 0.63
Leptin		0.337, *p* < 0.001	0.207, *p* = 0.006		0.345, *p* = 0.001	0.195, *p* = 0.070		0.347, *p* = 0.007	0.280, *p* = 0.033

Data presented as correlation coefficient (R) with *p* values

*FM%* fat mass percentage, *N* number, *PYY* peptide YY

In BF infants, leptin, ghrelin and ghrelin/PYY ratio at age 3 months correlated with the gain in FM% from 1 to 6 months (Table 3). In FF infants, however, only leptin at age 6 months correlated with the gain in FM% from 1 to 6 months as well as PYY at age 3 months.

The results in girls and boys were similar to those of the total group, but leptin in girls at age 6 months correlated with the change in FM% from 1 to 6 months (*R* = 0.379, *p* = 0.002), while it did not correlate in boys (*R* = 0.093, *p* = 0.33) (Supplemental Table).

### ARH and infant feeding at age 3 months

In addition, we investigated ARH at age 3 months in exclusively BF versus FF infants, thus without interference of infants-receiving mixed feeding or solid foods, in subgroup analyses. Median duration of breastfeeding in BF infants was 6.87 [4.65–10.02] months.

### Differences in ARH between BF versus FF infants at age 3 months

Ghrelin levels at age 3 months were lower and PYY levels were higher in BF infants compared to FF infants (*p* = 0.039 and < 0.001, respectively) (Table 4). The ghrelin/PYY ratio was lower in BF infants compared to FF infants (*p* = 0.002). Leptin levels at age 3 months tended to be lower in BF infants (*p* = 0.057).

**Table 4:** Ghrelin, PYY, ghrelin/PYY ratio and leptin levels at age 3 months in breastfed versus formula-fed infants

	Total group		*p* value	Boys		*p* value	Girls		*p* value
	Breastfeeding	Formula feeding		Breastfeeding	Formula feeding		Breastfeeding	Formula feeding	
	*n* = 158 (78 boys)	*n* = 89 (53 boys)		*n* = 78	*n* = 53		*n* = 80	*n* = 36	
Ghrelin (pg/ml)	51.9 [27.7–80.1]	67.0 [30.6–108.0]	**0.039**	52.4 [31.6–80.0]	65.0 [26.5–91.2]	0.47	50.6 [23.9–81.1]	81.0 [37.3–122.7]	**0.036**
PYY (pg/ml)	231.4 [178.2–314.5]	185.3 [151.0–245.5]	** < 0.001**	231.4 [177.2–306.8]	185.3 [150.1–231.1]	**0.001**	231.4 [176.6–326.5]	194.0 [150.8–262.8]	**0.027**
Ghrelin/PYY ratio	0.22 [0.10–0.36]	0.34 [0.15–0.61]	**0.002**	0.22 [0.12–0.39]	0.33 [0.15–0.61]	**0.038**	0.19 [0.10–0.35]	0.36 [0.14–0.55]	**0.015**
Leptin (pg/ml)	1090.5 [600.5–1776.9]	1417.0 [827.5–2191.7]	0.057	874.4 [457.8–1586.5]	1268.0 [660.0–1960.7]	0.091	1222.1 [771.8–1964.5]	1441.4 [954.8–2551.2]	0.16

Bold significance represent by *p* < 0.05

Data expressed as median [IQR]

*N* number, *PYY* peptide YY

### Correlations between ARH and human milk macronutrient content at age 3 months

In BF infants, ghrelin, PYY, ghrelin/PYY ratio and leptin levels at age 3 months did not correlate with human milk macronutrients (fat, energy, carbohydrate and protein) at age 3 months.

### Correlations between ARH and infant appetite at age 3 months

We investigated ARH levels in relation with infant appetite based on BEBQ scores. In BF infants, none of the ARH levels correlated with infant appetite.

In FF infants, PYY levels correlated inversely with infants getting full up easily (*R* = − 0.225, *p* = 0.039), indicating that higher PYY levels correlated with less easily getting full up during a feed. Higher ghrelin levels and ghrelin/PYY ratio tended to correlate with infants always demanding a feed (*R* = 0.205, *p* = 0.062 and *R* = 0.208, *p* = 0.059, respectively), indicating that higher ghrelin levels and ghrelin/PYY ratio correlated with less satiety. Leptin levels did not correlate with infant appetite outcomes of the BEBQ.

## Discussion

In a large group of healthy, term-born infants, we found that ghrelin levels and ghrelin/PYY ratio increased from 3 to 6 months, while PYY and leptin levels decreased. ARH levels were similar between boys and girls, except for a higher leptin in girls at 3 months. Leptin correlated with FM% at 3 and 6 months and the gain in FM% from 1 to 6 months, a critical window for adiposity programming, in BF and FF infants. In BF infants only, also ghrelin and ghrelin/PYY ratio correlated with the gain in FM% from 1 to 6 months. BF infants had lower ghrelin and higher PYY levels compared to FF infants at age 3 months. ARH levels did not correlate with human milk macronutrients. Regarding appetite, higher PYY levels in FF infants correlated with having more difficulty in getting full up during a feed, while a higher ghrelin level and ghrelin/PYY ratio tended to correlate with less satiety.

We present for the first time longitudinal levels of ghrelin, PYY and leptin during the first 6 months of life in healthy infants. These first 6 months after birth are considered a critical window for adiposity programming [9, 10]. Ghrelin levels increased significantly during the first 6 months of life, which is in line with a study from birth until 3 months [31], while PYY and leptin levels decreased. Our results complement current knowledge as other studies used cord blood to investigate leptin at birth [16, 17], a single measurement of ghrelin, PYY and leptin at 4 months [24] or a single measurement of ghrelin and leptin between 11 days and 22 months [23]. One study investigated leptin in multiple measurements until age 6 months, but only in a small group [32].

We also present ghrelin/PYY ratios during the first 6 months of life. Ghrelin/PYY might be a marker for orexigenic drive, as studies in subjects with Prader–Willi syndrome reported that subjects with hyperphagia due to PWS have hyperghrelinemia and attenuated PYY response to fat resulting in a high ghrelin/PYY ratio of 10 [5, 6]. We show that ghrelin/PYY ratio in healthy, term-born infants increased from age 3 to 6 months, but remained below 1.0.

Ghrelin levels at age 6 months correlated positively with fasting time; whereas, PYY levels correlated inversely with fasting time. This is in line with findings that ghrelin increases pre-prandially and decreases post-prandially and PYY levels act opposite with low levels in fasting state [33].

We investigated several factors that could potentially influence the levels of appetite-regulating hormones. Only leptin levels were different between boys and girls, with girls having higher levels at age 3 months, but not at age 6 months. Similar results have been reported for leptin levels at age 1, 4 and 6 months in a small group of infants [32] and at birth [15, 34].

Birthweight SDS did not correlate with ghrelin, PYY and leptin levels at age 3 and 6 months. One previous study reported an association between birthweight and leptin in cord blood with lower cord blood leptin associating with smaller size at birth [13]. Maternal pre-pregnancy BMI and weight gain during pregnancy did not correlate with ghrelin, PYY, ghrelin/PYY ratio and leptin levels. This is in contrast to a study showing that infants from mothers with high pre-pregnancy BMI (> 30 kg/m^2^) had higher levels of leptin at age 9 months, but ghrelin and PYY were not investigated [35]. The majority of mothers in our cohort had, however, a pre-pregnancy BMI below 25 kg/m^2^ and less than 10% had a BMI of > 30 kg/m^2^.

As the first 6 months of life are a critical window for adiposity programming [9, 10], we investigated ARH levels in relation with FM% and the gain in FM% during this period. Leptin at 3 and 6 months correlated with FM% at the same age, and with the gain in FM% from 1 to 6 months in the total group. In BF infants, ghrelin at 3 months did not correlate with FM% at the same age, but did correlate with FM% at 6 months, thus, 3 months later, suggesting that potential effects of ghrelin on FM% might reveal later while correlations between leptin levels and FM% are present at the same age. This might be explained by the fact that leptin is secreted by adipose tissue. In BF infants, also ghrelin and ghrelin/PYY ratio at 3 months correlated weakly with the gain in FM% during the critical window, while in FF infants, only PYY at age 3 months correlated with the gain in FM% from 1 to 6 months.

Studies with one leptin measurement either at birth [36], at age 4 months [24] or leptin measurements during the first 6 months in a small group of infants [37] have shown associations with the body composition. Two studies investigated leptin levels at birth or at age 6 months until childhood and associations with FM% and/or BMI in childhood [38, 39], but other ARH levels and measured FM% during the first 6 months were not investigated. We now show that leptin correlates not only with FM% at 3 and 6 months, but also with the gain in FM% in early life, which is of particular interest as we have previously shown that the gain in FM% during the first 6 months is associated with FM% at age 2 years [12].

We show that infants with exclusive breastfeeding versus formula feeding had different levels of ghrelin, PYY and leptin during the first 6 months of life. This is in line with our previous study in a smaller group of infants [25] and two other studies studying the first 4 months of life [23, 40]. FF infants had higher ghrelin levels, which stimulates intake, while PYY levels were lower, indicating less satiety. As a result, the ghrelin/PYY ratio was higher in FF infants, supporting a higher orexigenic drive in FF infants.

To the best of our knowledge, present study is the first one to present ghrelin, PYY, ghrelin/PYY ratio and leptin levels in relation to human milk macronutrient composition and infant appetite. In contrast to our hypothesis, ARH levels did not correlate with human milk fat and energy content in BF infants. We investigated human milk macronutrient composition and not the total daily intake and total daily macronutrient intake in BF infants, as it is difficult and laborious to measure the exact intake of human milk by 24 h infant weighing or deuterium oxide testing in large cohort studies in healthy infants. Future research could investigate if 24-h macronutrient intake will correlate with levels of ARH.

Infant appetite was investigated by the Baby Eating Behavior Questionnaire (BEBQ), a questionnaire for parents, at infant’s age of 3 months [30]. In BF infants, ARH did not correlate with infant appetite. In FF infants, however, higher PYY at age 3 months correlated with having more difficulty getting full up during a feed. Furthermore, higher PYY tended to correlate with less satiety. PYY decreases food intake and, as above-mentioned, PYY levels were lower in FF infants compared to BF infants, suggesting that they might indeed have less satiety. In addition, PYY correlated with the gain in FM% from 1 to 6 months in formula-fed infants, but not in breastfed infants, which suggest that early life PYY levels might contribute to the differences in body fat mass development between BF and FF infants.

PYY levels increase rapidly after food intake [2]. Our blood samples, however, were collected only in fasting state, which therefore should have lower PYY levels compared to non-fasting state and during feeding [41]. Stronger correlations are expected when investigating PYY peak levels in relation to infant appetite. When interpreting our results, one should take into consideration that the BEBQ is a subjective tool for infant appetite. Our exploratory results, however, emphasize the need for future research on associations of ARH, and specifically the ghrelin/PYY ratio, in relation to infant appetite during and after feeding.

The strength of this study is the availability of longitudinal blood samples during the critical window for adiposity programming in a large group of healthy infants. In addition, we obtained detailed body fat mass measures on the same day as infant blood collection was performed. We did, however, only show fasting ARH levels and were not able to collect samples during and after feeding as we could ethically not take multiple blood samples per infant. We could, therefore, not determine the PYY peak level after food intake.

In conclusion, we present appetite-regulating hormone trajectories in a large group of infants during the first 6 months of life, a critical window for adiposity programming. Ghrelin levels increased from 3 to 6 months, while PYY levels decreased. This results in an increase in ghrelin/PYY, suggesting more orexigenic drive over time. Leptin levels decreased in early life. ARH levels were similar between boys and girls, except for higher leptin levels in girls at 3 months. Formula-fed infants had higher ghrelin and lower PYY levels, thus a higher ghrelin/PYY ratio, suggesting that FF infants have higher orexigenic drive. Higher leptin levels correlated with higher FM% at 3 and 6 months and with a higher gain in FM% during the critical window for adiposity programming. In breastfed infants, ghrelin and ghrelin/PYY ratio also correlated with the gain in FM%, indicating that leptin and ghrelin levels might be involved in adiposity programming during early life.

## Supplementary Information

Below is the link to the electronic supplementary material.

Supplementary file1 (DOCX 14 KB)

## Data Availability

The data that support the findings of this study are available from the corresponding author upon reasonable request.
