# Supplementary material for: Appetite-regulating hormone trajectories and relationships with fat mass development in term-born infants during the first 6 months of life
Source: Eur J Nutr. 2021 Mar 25;60(7):3717–25. doi: 10.1007/s00394-021-02533-z (PMC8437841; doi:10.1007/s00394-021-02533-z)
Supplement: Supplementary file 1 — Supplementary file1 (DOCX 14 KB) [file 394_2021_2533_MOESM1_ESM.docx]

Supplemental Table. Correlations between appetite regulating hormones and FM% at age 3 and 6 months and the gain in FM% from 1-6 months in boys and girls.

|  | **Boys** (n=159) | | | **Girls** (n=138) | | |
| --- | --- | --- | --- | --- | --- | --- |
|  | **FM%  3 months** | **FM%  6 months** | **Δ FM%  1-6 months** | **FM%  3 months** | **FM%  6 months** | **Δ FM%  1-6 months** |
| **3 months** |  | | |  | | |
| Ghrelin | -0.070, p=0.39 | 0.098, p=0.23 | 0.067, p=0.42 | 0.060, p=0.48 | 0.159, p=0.075 | 0.146, p=0.11 |
| PYY | -0.0850, p=0.54 | 0.057,  p=0.49 | 0.025, p=0.76 | 0.008, p=0.93 | 0.071, p=0.43 | 0.123, p=0.17 |
| Ghrelin/PYY ratio | -0.057, p=0.48 | 0.056, p=0.50 | 0.057, p=0.49 | 0.035, p=0.69 | 0.112, p=0.22 | 0.065, p=0.48 |
| Leptin | **0.338, p<0.001** | **0.306, p<0.001** | **0.227, p=0.006** | **0.418, p<0.001** | **0.308, p<0.001** | **0.169, p=0.06** |
| **6 months** |  | | |  | | |
| Ghrelin |  | 0.018, p=0.85 | 0.030, p=0.75 |  | -0.083, p=0.52 | -0.060, p=0.64 |
| PYY |  | -0.010, p=0.92 | 0.124, p=0.19 |  | -0.087, p=0.50 | 0.032, p=0.80 |
| Ghrelin/PYY ratio |  | 0.012, p=0.90 | -0.06, p=0.95 |  | -0.040, p=0.75 | -0.060,  p=0.64 |
| Leptin |  | **0.268, p=0.003** | 0.093, p=0.33 |  | **0.456, p<0.001** | **0.379, p=0.002** |

Data presented as correlation coefficient (R) with p-values. FM%: fat mass percentage. N; number, PYY; peptide YY.
